# From mechanism to management: CEREMAST perspectives on the intersection of HαT and clonal mast cell disorders

**DOI:** 10.3389/falgy.2025.1674609

**Published:** 2025-11-11

**Authors:** Laura Polivka, Olivier Hermine, Julien Rossignol

**Affiliations:** 1 French Reference Center for Mastocytosis (CEREMAST), Paris Cité University, Necker—Enfants Malades University Hospital, AP-HP, Paris, France; 2 Department of Dermatology and Pediatric, Necker Enfants Malades, Assistance Publique (AP-HP), Paris Cité University, Paris, France; 3 Laboratory of Physiopathology and Treatment of Hematological Disorders, Imagine Institute, INSERM U1163, Paris, France; 4 Department of Hematology Cochin-Necker, Assistance Publique (AP-HP), Paris Cité University, Paris, France

**Keywords:** mast cells, mastocytosis, tryptase, *KIT*, hereditary alpha tryptasemia, diagnosis, pathophysiology

## Abstract

Since its initial description ten years ago, numerous studies have contributed to a better understanding of the role of hereditary alpha-tryptasemia (HαT) in the diagnosis and management of patients with clonal mast cell activation disorders (cMCADs). These studies have highlighted the high prevalence of HαT among cMCADs patients, the associated elevation in baseline serum tryptase levels—which can influence both diagnosis and disease monitoring—and distinct clinical features, notably an increased risk of severe anaphylaxis. As a result, screening for HαT has become an integral part of the diagnostic work-up in patients with cMCADs. However, several key questions remain unresolved: Why is HαT more prevalent among cMCADs patients? How can we accurately distinguish between HαT and cMCADs during the diagnostic process? And how does the presence of this genetic trait influence the clinical management of cMCADs? In this article, we present the position and clinical approach of the French National Reference Center for Mastocytosis (CEREMAST).

## 1 Introduction

**Figure 1:**
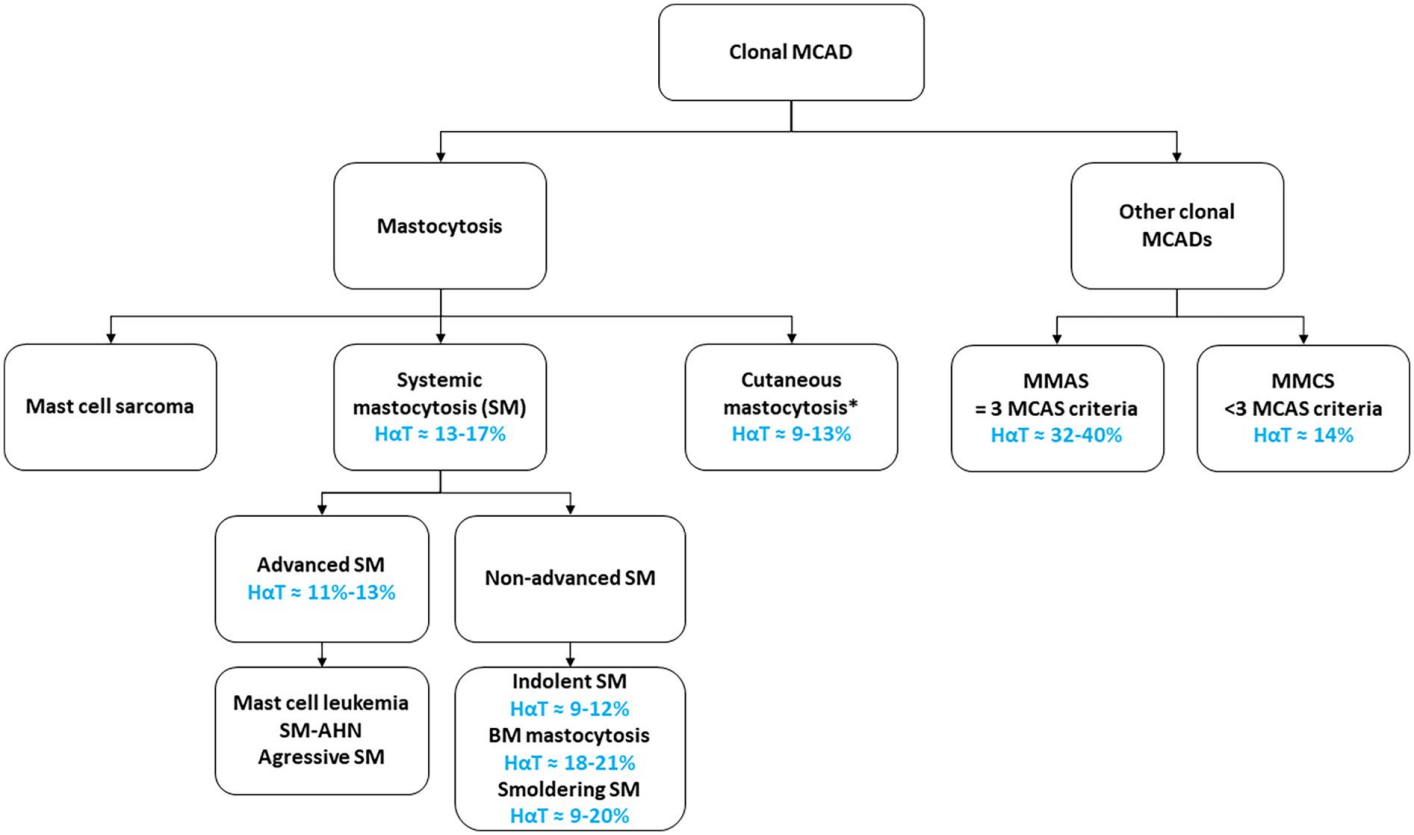
Hαt prevalence according to clonal mast cell activation disorders subtypes. HαT prevalence in general population ≈ 4%–6%. HαT prevalence in cMCADs patients was reported in the following references (7–10). HαT, hereditary α-tryptasemia; MCAD, mast cell activation disorder; SM, systemic mastocytosis; BM, bone marrow; SM-AHN, SM with an associated hematologic neoplasm; MCAS, mast cell activation syndrome; MMAS, monoclonal MCAS; MMCS, monoclonal mast cells with clinical significance. Mastocytosis is classified according to the WHO and ICC classifications. Other clonal MCADs require the presence of signs of clonality, such as an activating *KIT* mutation and/or aberrant expression of CD2 and/or CD25 and/or CD30 on mast cells. MCAS diagnosis requires the presence of all three of the following diagnostic criteria: (i) Typical symptoms consistent with MC activation (ii) a ≥20% increase plus an absolute increase of ≥2 ng/mL in serum tryptase during an acute episode compared with basal serum tryptase, and (iii) a significant response to MC-stabilizing agents. *In adults’ patients.

Mast cell activation disorders (MCADs) encompass a group of diseases characterized by abnormal accumulation and/or activation of mast cells (MCs) in tissues (1–3). These disorders are classified based on the presence or absence of evidence of clonality (e.g., *KIT* gene mutations, aberrant expression of CD2 and/or CD25 and/or CD30 on MCs). Non-clonal MCADs are primarily represented by MC activation syndrome (MCAS), which requires the presence of all three of the following diagnostic criteria: (i) typical symptoms consistent with MC activation, (ii) a ≥20% increase plus an absolute increase of ≥2 ng/mL in serum tryptase during an acute episode compared with serum basal tryptase (sBT), and (iii) a significant response to MC-stabilizing agents (2, 4–6). Clonal MCADs include a large group of disorders, the most common and well-known of which is mastocytosis (Figure 1). Mastocytosis is classified according to the World Health Organization (WHO) and International Consensus Classification (ICC) into three major subtypes (11–16): cutaneous mastocytosis (CM), MC sarcoma, and systemic mastocytosis (SM). SM is further subdivided into non-advanced forms, which include indolent SM (ISM), bone marrow mastocytosis (BMM), and smoldering SM (SSM); and advanced SM (Adv-SM), including MC leukemia (MCL), aggressive SM (ASM), and SM associated with another hematologic neoplasm (SM-AHN) which is the most frequent subtype of Adv-SM. Other recently described clonal MCADs are defined by the presence of clonal markers without meeting the full diagnostic criteria for mastocytosis. These cMCADs include: monoclonal MCAS (MMAS), diagnosed when all three MCAS criteria are met alongside evidence of MC clonality, and monoclonal MCs with clinical significance (MMCS), in which clonal MCs are present in the BM but at least one MCAS diagnostic criterion is absent (17).

A genetic trait known as hereditary α-tryptasemia (HαT) was first described approximately ten years ago by Lyons et al. (18). HαT is caused by duplication or amplification of the alpha-1 allele of the *TPSAB1* gene and is associated with a sBT level greater than 8 ng/mL. Initially, the description was limited to the genetic and clinical characterization of several families, and the potential for HαT to modulate clinical manifestations or influence daily practice through systematic screening was not anticipated. However, subsequent studies have demonstrated that these genotypes are highly prevalent in Western populations, with a frequency ranging from 4.4% to 5.6% depending on the cohort, making HαT the most common cause of elevated sBT (7–19). The prevalence of this genotype in other continents, particularly in Asia and Africa, as well as individuals’ ethnic backgrounds within Western populations remains to be thoroughly investigated. *TPSAB1* genotyping using droplet digital PCR (ddPCR) for the detection of HαT has now become a routine diagnostic tool in many countries and may be included in the diagnostic workup when MCADs are suspected. Nonetheless, its exact role within diagnostic algorithms is not yet fully defined. Furthermore, HαT prevalence has been reported to be higher in patients with cMCADs compared to the general population, and its association with cMCADs has been linked to clinical and biological features (7–10). However, the clinical impact of HαT on the management of patients with MCADs remains a subject of debate and lacks consensus.

Here, we present our perspective from the French National Reference Center for Mastocytosis (CEREMAST), which manages over 2,500 patients with MCADs, on the role and implications of HαT in the clinical management of patients.

## 2 Hereditary alpha-tryptasemia: bridging the gap between genotype and clinical practice

### 2.1 Potential mechanisms linking HαT to monoclonal mast cell proliferation

#### 2.1.1 Better diagnostic accuracy or underlying pathophysiological association?

One of the earliest and most consistently reported features associated with HαT is its increased frequency among patients with cMCADs. Indeed, the prevalence of HαT in this population has been estimated to range from 12.2% to 17.2%. Interestingly, this prevalence is not uniform across all cMCADs subtypes. For example, the frequency of HαT was reported as 12% in CM, 12% in ISM, but 20% in BMM, and 33% in MMAS—the latter two being forms of cMCADs without cutaneous involvement (7).

This overrepresentation of HαT in non-cutaneous forms of cMCADs has raised the hypothesis of a possible improved detection of clonal MCADs in HαT carriers (Table 1). Indeed, HαT has been consistently associated with (i) increased baseline serum tryptase levels in both healthy individuals and those with cMCADs, and (ii) an increased risk of anaphylaxis, particularly severe episodes, in patients with clonal MCADs. Thus, one can hypothesize that HαT carriers may be more likely to be diagnosed with cMCADs due to their distinctive clinical and biological features—especially in non-cutaneous forms, which are more challenging to diagnose. Indeed, in these forms, diagnostic algorithms heavily rely on elevated serum tryptase levels and the presence and severity of anaphylaxis.

**Table 1:** Potential mechanisms linking HαT to clonal mast cell activation disorders.

Hypothesis	Overdiagnosis of cMCADs in HαT + vs. HαT− patients	HαT as a risk factor of cMCADs
Supporting arguments	Higher prevalence of HαT observed in BMM and MMAS which are associated with anaphylaxis	Increased prevalence of HαT in Adv-SM
Proposed mechanisms	Association of HαT with elevated serum basal tryptase and higher risk of anaphylaxis → increased likelihood of cMCADs diagnosis	Increased MC numbers in the gastrointestinal tract; abnormal bone marrow MC morphology (spindle shape, hypogranulation)
Potential clinical implications	Up to two-thirds of cMCADs may be underdiagnosed in HαT− individuals True prevalence of cMCADs might be underestimated—cMCADs potentially not a rare disease?	Adaptation of cMCADs diagnostic algorithms (e.g., osteoporosis, hymenoptera anaphylaxis…) according to HαT status?

HαT, hereditary α-tryptasemia; cMCADs, clonal mast cell activation disorders; Adv-SM, advanced systemic mastocytosis; BMM, bone marrow mastocytosis; MMAS, monoclonal mast cell activation syndrome.

On the other hand, a higher prevalence of HαT has also been found in patients with Adv-SM (7). These patients are typically diagnosed due to the presence of C findings (in particular cytopenia, ascites, osteolytic lesions not attributable to osteoporosis, weight loss with malabsorption) or through evaluation of a concurrent hematological neoplasm, and rarely due to anaphylaxis. Moreover, baseline tryptase levels are significantly higher in Adv-SM compared to non-Adv-SM, making HαT status a less influential factor in triggering diagnosis. This raises the alternative hypothesis that HαT may also be a risk factor—at least partially—for the development of cMCADs (Table 1). Although no mechanistic data have yet been published, several specific features have been observed in HαT+ patients without clonal MCADs. For instance, patients with HαT and irritable bowel syndrome have shown increased MC counts in gastrointestinal biopsies compared with HαT− patients (20, 21). Additionally, in HαT+ patients with MCAS, bone marrow MCs may display abnormal morphology, including spindle-shaped cells and hypogranulation, reminiscent of those seen in mastocytosis (22). Taken together, the increased MC burden and atypical morphology in HαT+ individuals might enhance the likelihood of acquiring somatic *KIT* mutations, thus promoting the development of cMCADs.

#### 2.1.2 Clinical implications of the two hypotheses

These two-hypothesis mentioned above could have an impact on patient management. It is conceivable that one, the other, or even both hypotheses (better diagnosis and increased risk of cMCADs occurrence, Table 1) may be valid. In the case of improved detection of cMCADs among HαT+ patients, this would imply a potential underdiagnosis among HαT− individuals—potentially affecting up to two out of three patients. To reduce underdiagnosis in HαT− patients, broader training and awareness, as well as the wider implementation of diagnostic tools for cMCADs—such as detection of the *KIT* D816V mutation by ddPCR—should be considered. In addition, considering recent data suggesting a prevalence of mastocytosis approximately 1 in 3,500 individuals, the true prevalence of cMCADs may in fact be twice as high (23). This would challenge the current classification of cMCADs as a rare disease.

Conversely, if there is a true pathophysiological link and an estimated threefold increased risk of developing cMCADs in HαT+ individuals, this population may receive particular attention. While the overall risk of cMCADs remains low, diagnostic algorithms may need to be revisited to distinguish between HαT+ and HαT− patients. For instance, in the evaluation of early-onset osteoporosis or hymenoptera venom-induced anaphylaxis there may be a potentiallyincreased use of bone marrow investigations in HαT+ individuals. Finally, under this hypothesis, since the prevalence of clonal MCADs among individuals with HαT remains low—estimated between 1 in 1,100 and 1 in 3,500—we do not believe that genetic counseling for reproductive purposes or routine family screening is warranted at this time.

### 2.2 Diagnostic challenges in distinguishing HαT from other clonal mast cell activation disorders

As mentioned above, HαT is the most common cause of elevated sBT, with levels directly correlated to the number of α-tryptase gene copies. While most individuals with HαT have sBT levels between 8 and 15 ng/mL, those carrying three or more copies of the α-allele may present with significantly higher levels as observed in Adv-SM. It is therefore critical to interpret sBT in the context of HαT status when evaluating patients for suspected cMCADs and sBT >8 ng/mL. In patients with cMCADs and cutaneous lesions (i.e., ISM or CM), the diagnosis of mastocytosis in the skin is typically confirmed through a skin biopsy, meaning that identification of HαT is unlikely to lead to misdiagnosis. However, the diagnostic challenge arises in the absence of cutaneous lesions—particularly in suspected cases of cMCADs such as BMM, MMCS, or MMAS. In such cases, bone marrow evaluation (including aspirate and biopsy) remains the only method to definitively confirm or rule out the diagnosis, but it cannot be systematically performed in all patients. Therefore, it is crucial to estimate the probability of cMCADs to better interpret the significance of identifying HαT in a given patient. In certain contexts, the presence of HαT may support the exclusion of a cMCADs diagnosis, thereby helping to avoid invasive procedures. However, in other scenarios, HαT does not rule out cMCADs, and clinicians should be cautious not to prematurely dismiss the diagnosis.

In recent years, droplet digital PCR (ddPCR) and allele-specific oligonucleotide qPCR (ASOqPCR) for detection of the *KIT* D816V mutation in peripheral blood have become important screening tools for cMCADs. However, in patients without skin involvement, these assays have significantly lower sensitivity in blood than in bone marrow—estimated around 50%–60% (24, 25). Therefore, a negative peripheral blood *KIT* result, even in the context of confirmed HαT, should not be used to systematically exclude the diagnosis of cMCADs. As the saying goes: “Don't let the obvious blind you to what's behind”. Overall, we propose three clinical scenarios to interpret the presence or absence of HαT, based on the estimated probability of cMCADs without skin lesion in patients with sBT >8 ng/mL.

Given the increased awareness and more frequent ordering of tryptase tests by physicians in allergology, internal medicine, and general practice, we are seeing a growing number of patients with moderately elevated sBT levels, without clear evidence of cMCADs. These include patients presenting with chronic or recurrent symptoms of mast cell activation, but without a history of severe anaphylaxis (particularly to hymenoptera venom or idiopathic), without early-onset severe trabecular osteoporosis (the probability of cMCADs can be assessed using the recently published scoring system in this context) or unexplained chronic hypereosinophilia (26–30). In such cases, first we ensure that no skin lesions of mastocytosis, lymphadenopathies, hepatomegaly, splenomegaly are present, and we test for the *KIT* D816V mutation in peripheral blood and assess for HαT. If HαT is present and the sBT level is consistent with the number of α-allele copies of *TPSAB1*, no further investigations are pursued. However, if the sBT is higher than expected, bone marrow evaluation is performed.

Conversely, when there is a clear clinical presentation consistent with cMCADs, we systematically conduct bone marrow investigations, regardless of the presence or absence of HαT. For example, HαT+ patients with hymenoptera venom allergy and a REMA score ≥2 are evaluated even in the absence of a detectable *KIT* mutation in blood. Similarly, patients with unexplained chronic hypereosinophilia and/or early-onset severe trabecular osteoporosis may undergo bone marrow investigation, regardless of HαT status.

### 2.3 Management strategies for patients with both HαT and monoclonal mast cell activation disorders

#### 2.3.1 Hαt and the interpretation of serum baseline tryptase levels

HαT has significantly influenced the management strategy of patients with cMCADs. During the diagnostic work-up, accounting for HαT when interpreting sBT level has become critical. Indeed, the minor criterion defined by the WHO and ICC requires a sBT level >20 ng/mL, but this threshold must be interpreted in the context of the number of α-tryptase gene copies. For this purpose, we routinely use the online calculator (https://bst-calculater.niaid.nih.gov/), which helps determine whether the sBT level is elevated beyond what would be expected based on the individual's α-tryptase copy number—and thus whether the minor WHO/ICC criterion is truly fulfilled. Beyond diagnosis, α-tryptase copy number should also be considered in the assessment of therapeutic response. In non-advanced mastocytosis, where treatment endpoints primarily focus on symptom control and quality of life, changes in sBT levels may reflect a reduction in mast cell burden; however, absolute sBT levels are not critical for response evaluation. However, in Adv-SM, where response assessment relies on objective laboratory parameters, interpretation of sBT levels according to α-tryptase gene dosage is critical. Indeed, response criteria for AdvSM, in addition to pathological response, biological response includes both the variant allele frequency (VAF) of *KIT* D816V (assessed via ddPCR or ASOqPCR) and sBT. In patients with HαT, normalization of tryptase levels is not achievable and must therefore be interpreted in the context of the expected sBT based on α-copy number, along with the reduction in *KIT* D816V VAF.

#### 2.3.2 Hαt and therapeutic management of cMCADs patients

To date, no international recommendations have been issued regarding the therapeutic management of patients with cMCADs based on the presence or absence of HαT. However, available literature supports a personalized approach for these patients. Indeed, individuals with both HαT and cMCADs are at increased risk of anaphylaxis, particularly from hymenoptera venom, and notably with a higher likelihood of grade IV reactions. Although, in general, the routine prescription of epinephrine auto-injectors for primary prevention of hymenoptera venom–induced anaphylaxis is not recommended for all cMCADs patients, we now routinely prescribe it to patients with both HαT and cMCADs, especially when they are likely to spend time in remote areas with limited access to emergency care. To date, no therapeutic studies have demonstrated a differential response based on HαT status—whether to mediator-targeted therapies or tyrosine kinase inhibitors. As such, we do not currently modify symptomatic treatment protocols based on HαT status.

## 3 Discussion

The discovery and emerging understanding of HαT has introduced a new layer of complexity in the diagnostic and clinical management of patients with cMCADs. This genetic trait, long overlooked, now plays a central role in interpreting both diagnostic criteria and therapeutic response thresholds. However, several questions remain unanswered—ranging from pathophysiological mechanisms to diagnostic strategies and therapeutic management.

Firstly, its high prevalence in patients with cMCADs raises two non-mutually exclusive hypotheses: overdiagnosis in HαT+ patients compared with HαT− patients, driven by HαTrelated biomarkers and symptoms, or a genuine pathophysiological predisposition to clonal MC proliferation. This pathophysiological question is not merely rhetorical, as its implications could be significant both from an epidemiological standpoint and for the development of diagnostic algorithms tailored to HαT status. Robust scientific studies are therefore urgently needed to disentangle these possibilities. Although longitudinal follow-up of individuals based on their HαT status may seem appropriate, such studies are likely to face statistical challenges due to the low incidence of cMCADs, even among patients with HαT. In parallel, investigations into the prevalence of the *KIT* D816V mutation in the general population—as seen in clonal hematopoiesis of indeterminate potential—could also provide valuable insights into the underlying mechanisms.

From a diagnostic standpoint, screening for HαT has become essential in the work-up of suspected cMCADs. Indeed, identifying HαT in cases of low clinical suspicion for cMCADs can help avoid unnecessary invasive procedures such as bone marrow aspiration and biopsy, which would have previously been performed. However, the detection of HαT can also be misleading, as clinicians may prematurely halt the diagnostic work-up upon its discovery. This is particularly relevant in patients without cutaneous involvement, where the detection of the *KIT* D816V mutation using blood-based ddPCR is increasingly utilized, despite its sensitivity being only about half that of bone marrow-based assays. This distinction is critical, as misdiagnosing cMCADs patients with coexisting HαT could have serious consequences, given their increased risk of anaphylaxis and the availability of effective treatment, such as long-term venom immunotherapy. New blood-based screening techniques, such as highly sensitive tandem PCR [recently presented at the ASH congress (31)], appear promising and could further improve diagnostic accuracy and sensitivity while reducing the need for invasive procedures.

Lastly, from a therapeutic standpoint, HαT patients with cMCADs may benefit from more proactive preventive strategies, such as the systematic prescription of epinephrine auto-injectors. On the other hand, there is currently no evidence to support symptomatic or curative treatment modifications specifically based on HαT status. However, systematic screening for HαT in future clinical trials—including both non-advanced and advanced subtypes of mastocytosis—appears crucial, as HαT could potentially influence treatment response and might eventually guide personalized therapeutic strategies depending on the type of medication used (e.g., biologics, tyrosine kinase inhibitors).

## Data Availability

The original contributions presented in the study are included in the article/Supplementary Material, further inquiries can be directed to the corresponding author.
